# Enhancing Bioactivity and Mechanical Properties of Nano-Hydroxyapatite Derived from Oyster Shells through Hydrothermal Synthesis

**DOI:** 10.3390/nano14151281

**Published:** 2024-07-30

**Authors:** Shih-Ching Wu, Hsueh-Chuan Hsu, Wen-Hui Wu, Wen-Fu Ho

**Affiliations:** 1 Department of Dental Technology and Materials Science, Central Taiwan University of Science and Technology, Taichung 406053, Taiwan; scwu@ctust.edu.tw (S.-C.W.); hchsu@ctust.edu.tw (H.-C.H.); 2 Department of Chemical and Materials Engineering, National University of Kaohsiung, Kaohsiung 811726, Taiwan

**Keywords:** hydroxyapatite, oyster shells, hydrothermal reaction, bioactivity, sintering, cell culture, microhardness

## Abstract

Nano-hydroxyapatite (nHA) demonstrates favorable biological activity, cell adhesion, cell proliferation, and osteoconductivity, making it highly valuable in biomedicine. It is extensively used as a bone substitute and in bone transplantation within the dental and orthopedic fields. This study employed oyster shells as a calcium source to synthesize nHA at 150 °C with various hydrothermal reaction durations (10 min, 1 h, 6 h, and 12 h). As a control, HA synthesized via a wet precipitation method for 1 h at room temperature was utilized. Subsequent material analyses, including XRD, FE-SEM, FTIR, and ICP-MS, were conducted, followed by comprehensive evaluations of the bioactivity, cell attachment, cell proliferation, and sintering properties of the synthesized nHA. The results indicated that nHA synthesized through the hydrothermal reaction produced nanoscale crystals, with the aspect ratio of nHA particles increasing with the duration of hydrothermal treatment. Notably, rod-like nHA particles became prominent with hydrothermal durations exceeding 6 h. nHA particles derived from oyster shells contained carbonate and trace elements (Na, Mg, K, and Sr), similar to constituents found in human hard tissue such as bone and teeth. The immersion of nHA synthesized at 150 °C for 1 h (HT2) in simulated body fluid (SBF) for 28 d led to the formation of a bone-like apatite layer on the surface, indicating the excellent bioactivity of the synthesized nHA. The cell culture results revealed superior cell attachment and proliferation for nHA (HT2). Following the sequential formation and sintering at 1200 °C for 4 h, HT2 ceramics exhibited enhanced microhardness (5.65 GPa) and fracture toughness (1.23 MPa·m^0.5^), surpassing those of human tooth enamel.

## 1. Introduction

Due to demographic changes and management strategies, fractures are becoming an increasing burden on healthcare resources. The global incidence of fractures is 99 cases per 100,000 people annually [1]. Hydroxyapatite (HA) is well regarded for its bioactivity, cell adhesion, cell proliferation [2], and osteoconductivity [3], making it a prominent material in biomedical applications. Due to its ability to form bonds with bone tissue, HA is extensively utilized as a bone replacement and graft material [4,5]. Additionally, HA is employed in bioactive coatings [6,7], protein delivery media [8], drug carriers [9], and for the purification of nucleic acids during chromatographic analysis [10]. As the principal inorganic component of skeletal tissue, HA is often extracted from natural biological sources such as fish bone, goat bone, cattle bone, and chicken bone, with bovine bone being the most commonly used [11,12,13]. Bovine bone is widely used for HA production due to its abundance from the meat industry, similar mineral composition to human bone, and proven biocompatibility and efficacy in bone regeneration applications.

Besides extraction from natural biological sources, HA can also be synthesized chemically. Common synthetic methods include aqueous precipitation, microwave irradiation, and hydrothermal reactions [14,15,16]. The hydrothermal method is particularly notable for its simplicity and reproducibility, allowing for control over the crystal phase, size, and crystallinity of HA by adjusting parameters such as the temperature, time, solution pH, and pressure [17,18].

With the increasing interest in recycled materials, researchers have explored the synthesis of HA from natural wastes. Alif et al. [19] used freshwater clam shell waste to synthesize HA via a hydrothermal method, involving the calcination of the shells followed by humid conditioning for one week to obtain calcium hydroxide before adding ammonium dihydrogen phosphate (NH_4_H_2_PO_4_). Ferro et al. [20] utilized eggshells and cuttlefish bones to prepare HA through a mechanochemical route. Oyster shells, composed mainly of calcium carbonate (approximately 96%) [21], are also an excellent calcium source for HA synthesis. The resulting HA contains beneficial trace elements (Na, Mg, Sr, etc.) derived from the oyster shells. Given that the Ca^2+^ in HA can be substituted with various metal cations, research has focused on synthesizing HA with trace metal elements such as Mg, Sr, Ba, and Cu [22,23].

In recent years, the synthesis of HA from waste biomass has gained increasing attention. This approach effectively utilizes waste resources, reduces environmental pollution, and lowers the production costs. Oyster shells, rich in Ca and readily available, have become an ideal raw material for HA synthesis. Several studies have reported the successful synthesis of HA using oyster shells, demonstrating the feasibility and advantages of this method. These studies highlight the potential of oyster shell-derived HA in biomedical applications, showing that, through appropriate chemical and thermal treatments, high-purity and high-crystallinity HA can be extracted from oyster shells for use in bone grafts, bone repair, and other medical fields. Ho et al. [24] utilized oyster shells to produce nHA powder through liquid-phase precipitation, which was then used to create biphasic calcium phosphate (BCP) bioceramics. The results showed that shorter, high-temperature sintering yielded BCPs with hierarchical pore structures, enhancing their biocompatibility and promoting the differentiation of induced pluripotent stem cells into osteoblasts. Wu et al. [25] synthesized HA powder from oyster shell powders and dicalcium phosphate dihydrate using ball milling and heat treatment. The resulting HA exhibited good crystallinity (96.3%) and high phase purity, containing trace elements such as Mg and Sr.

Biological HA in animal bones exists as nanoscale crystals. Nanosized HA (nHA) exhibits superior protein adsorption, osteoblast adhesion [26], cell proliferation, and bioactivity [27,28,29]. Furthermore, nHA demonstrates better densification upon forming and sintering [30].

This study investigates the synthesis of nHA using oyster shells as a calcium source via the hydrothermal method. The effects of different hydrothermal reaction times (10 min, 1 h, 6 h, and 12 h) on the characteristics of nHA were examined. Additionally, the sintering properties, bioactivity, cell attachment, and cell proliferation of nHA were explored.

## 2. Materials and Methods

After cleaning, the oyster shells were calcined at 1100 °C for 4 h. The calcined shells were then pulverized into fine powders and sieved through a 200-mesh sieve. To prepare the calcium-containing solution, 2.13 g of the calcined oyster shell powder was added to 20 mL of deionized water and stirred for 8 h at a speed of 300 rpm and a temperature of 50 °C. Additionally, 2.976 g of (NH_4_)_2_HPO_4_ was dissolved in 15 mL of deionized water. Once fully dissolved, this solution was poured into the calcium-containing solution. The pH value was adjusted to 10 using an aqueous solution of (NH_4_)OH. The resulting solution was then transferred into a Teflon bottle, which was sealed and placed in a stainless steel autoclave for hydrothermal treatment at 150 °C for various durations (10 min, 1 h, 6 h, and 12 h).

**Figure 1:**
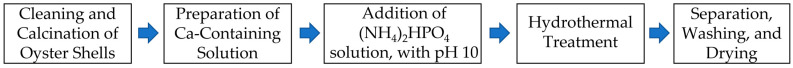
Schematic diagram of the HA synthesis process from oyster shells.

The hydrothermally treated suspension was separated using a centrifuge and repeatedly washed with deionized water ten times. The washed suspension was then dried in an oven at 70 °C and ground into powders. The HA samples synthesized by hydrothermal treatment for 10 min, 1 h, 6 h, and 12 h are labeled HT1, HT2, HT3, and HT4, respectively (Table 1). For comparison, HA prepared by the wet precipitation method at room temperature for 1 h was used as a control group and designated as WP. The schematic diagram of the HA synthesis process from oyster shells in this experiment is shown in Figure 1.

An X-ray powder diffractometer (XRD, D8 Advance, Bruker, Berlin, Germany) was utilized for crystalline phase analysis, operating with Cu Kα radiation generated at 40 kV and 40 mA. The diffraction angles (2θ) for each sample were measured from 20° to 50° at a scan speed of 5°/min and a step size of 0.02°. The crystallite size of the synthesized HA was calculated using the (002) diffraction peak and the Scherrer formula [31]:

(1) $$D=\frac{0.9\lambda }{\mathrm{FWHM}\;\cos\theta }$$

where λ is the X-ray wavelength (0.15406 nm), FWHM (rad) is the full width at half maximum of the (002) reflection, θ (degrees) is the Bragg angle, and D (nm) is the crystallite size. The crystallinity (Xc) of the synthesized HA was calculated using the following formula [32]:

(2) $$X_{c}=1-\frac{V_{112/300}}{I_{300}}$$

where V_112/300_ is the intensity of the hollow between the (112) and (300) diffraction peaks, and I_300_ is the intensity of the (300) diffraction peak.

The HA powders were observed using a field emission scanning electron microscope (FE-SEM, S-4800, Hitachi, Tokyo, Japan). FE-SEM images were employed to calculate the length and width of the HA particles. The electron acceleration voltage employed to obtain the SEM images was 10 kV. Additionally, the samples were coated with a thin layer of gold before imaging to enhance the conductivity and improve the image quality. Six images were taken for each sample, with measurements taken from 10 particles in each image to determine the average particle length and width.

The functional groups of the prepared HA were analyzed using a Fourier transform infrared (FT-IR) spectrometer (Cary 630, Agilent, Santa Clara, CA, USA). HA and KBr were mixed uniformly at a weight ratio of 1:150 and pressed into translucent sheets for testing. Measurements were conducted over the wavenumber range of 600–4000 cm^−1^. Additionally, an inductively coupled plasma mass spectrometer (ICP-MS, 7500ce, Agilent, Santa Clara, CA, USA) was used to measure the elemental content of the oyster shell powder and the prepared HA powder.

The apatite-forming ability on the surface of the sample was observed by immersing it in simulated body fluid (SBF) to evaluate its bioactivity. The HA powder was pressure-molded into wafer discs with a diameter of 13 mm and a thickness of 1 mm, followed by heat treatment at 400 °C for 2 h with a heating rate of 1.5 °C/min. The SBF was prepared according to the formula proposed by Kokubo and Takadama [33]. The sample was placed in a beaker filled with 50 mL of SBF solution, covered with plastic wrap, and incubated in a constant-temperature water bath at 37 °C for 1, 7, 14, and 28 days. The SBF solution was replaced every 2 days to maintain the ion concentration. Three test samples were prepared for each condition. After immersion, the samples were dried in an oven at 70 °C and analyzed using SEM and HR-XRD.

Human osteoblast MG-63 cell suspensions were used for culture and cell viability evaluations. The HA powders were fixed on glass slides with carbon tape, sterilized with high-pressure steam at 121 °C for 1 h, and then placed in a 24-well culture plate. Cells were seeded (the initial cell density was 1 × 10^4^ cells/mL), and pure DMEM culture medium was added to each well to a total volume of 2 mL. The culture plate was incubated in a 5% CO_2_ atmosphere at 37 °C for 1 and 7 days, with the culture medium changed every 2 days. After cell culture, the samples were washed with PBS and transferred to a new 24-well culture plate. WST-1 reagent was added and the samples were incubated for 4 h. Subsequently, 100 µL of the reagent was transferred to a 96-well culture plate, and the absorbance at 450 nm was measured using an ELISA reader (Multiskan GO, Thermo Scientific, Waltham, MA, USA). Additionally, glutaraldehyde was used to fix the cells, which were then sequentially dehydrated and dried at critical points for the observation of the cell distribution and attachment by SEM.

The HA powders were uniaxially press-molded into green compacts with a diameter of 13 mm and a thickness of 3 mm, followed by a two-stage heat treatment. In the first stage, the temperature was raised to 500 °C for 6 h to burn off the binder and lubricating oil, followed by furnace cooling to room temperature. In the second stage, the temperature was maintained at 800 °C for 4 h and then increased to 1200 °C for 4 h for sintering, followed by air cooling. The heating rate for each stage was 5 °C/min. The sintered samples were analyzed for their crystalline phases using XRD and compared with the International Centre for Diffraction Data (ICDD) using JCPDS card No. 72-1243 for HA, No. 06-0426 for β-TCP, and No. 09-0348 for α-TCP.

The Archimedes method was employed to measure the apparent density of the samples, calculated using the following formula:

(3) $$\rho_{a}=\frac{W_{1}}{{(W}_{3}-W_{2}{)/\rho }_{\mathrm{water}}}$$

where ρ_a_ (g/cm^3^) is the apparent density of the sample, W_1_ (g) is the dry weight, W_2_ (g) is the weight measured in deionized water, W_3_ (g) is the saturated weight, and ρ_water_ is the density of water (1 g/cm^3^). The relative density of the sample was then calculated using the formula

(4) $$\rho_{r}=\frac{\rho_{a}}{\rho_{th}}\times 100\%$$

where ρ_r_ (%) is the relative density of the sample, and ρ_th_ (g/cm^3^) is the theoretical density of the sample.

The samples were sequentially ground with #400, #800, #1200, #1500, and #2000 sandpapers, followed by polishing with a 1 μm diamond lapping film. Subsequently, the samples were subjected to thermal corrosion at 1100 °C for 15 min. The grain sizes and pores of the samples were observed using SEM. The grain size was measured according to the intercept method specified in ASTM E112-96 [34]. Additionally, after hot corrosion, the samples were subjected to micro-indentation with a micro-Vickers hardness tester (HMV-2, Shimadzu, Tokyo, Japan), using a load of 1.96 N and a dwell time of 10 s, and the cracks were examined by SEM.

The indentation test was used to calculate the microhardness and fracture toughness. The microhardness was measured according to ASTM C1327-15 [35], and the fracture toughness was determined using the formula summarized by Niihara et al. [36]. Hardness values were measured at least at five different positions on each sample, with three samples tested for each condition, ensuring a minimum of 15 data points per condition for average value calculation.

(5) $$\mathrm{HV}=1.8544\frac{W}{d^{2}}$$

(6) $$K_{\mathrm{Ic}}=0.203{\left(\frac{c}{a}\right)}^{1.5}HV{\left(a\right)}^{0.5}$$

where W (N) is the load, HV (GPa) is the hardness value, d (μm) is the average diagonal length of the indentation, K_Ic_ (MPa·m^0.5^) is the fracture toughness, c (μm) is the average crack length of the indentation, and a (μm) is the average value of half the diagonal length of the indentation.

The graphs (XRD, FT-IR, and WST-1 assay) were generated with the Origin 9.0 software. The cell culture data were analyzed with Student’s *t*-test. Statistical significance was set at a *p*-value of less 0.05.

## 3. Results and Discussion

### 3.1. Characteristics of nHA Prepared under Various Hydrothermal Reaction Times

**Figure 2:**
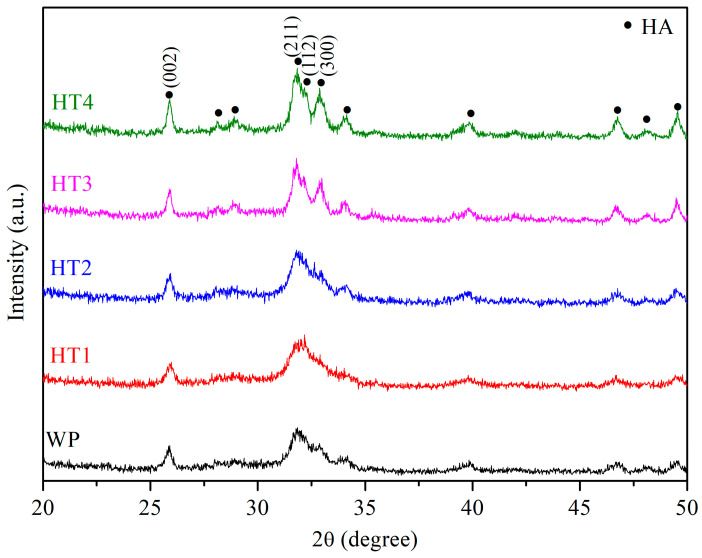
XRD diffraction patterns of nHA synthesized by wet precipitation method (WP) and hydrothermal method at various reaction times at 150 °C (HT1: 10 min; HT2: 1 h; HT3: 6 h; HT4: 12 h).

Figure 2 presents the XRD diffraction patterns of the nHA synthesized by the wet precipitation method (WP) and hydrothermal method at various reaction times at 150 °C (HT1, HT2, HT3, and HT4). All conditions yielded single-phase nHA (JCPDS file No. 09-0432), with no other phases or residual raw materials observed. Notably, the diffraction peaks of WP, HT1, and HT2 are broader, whereas those of HT3 and HT4 are sharper, which can be attributed to variations in crystallinity and grain size. Generally, lower crystallinity and smaller grain sizes result in broader diffraction peaks [37,38]. As the hydrothermal reaction time increases, the crystallinity of nHA improves, leading to slightly sharper diffraction peaks. Table 2 displays the crystallinity of the nHA synthesized under each condition, showing an increase in crystallinity from 26.04% for HT1 to 54.12% for HT4 with longer hydrothermal reaction times. At the same reaction time of 1 h, WP exhibits lower crystallinity compared to HT2, indicating that the hydrothermal reaction enhances the crystallinity. The Scherrer formula was employed to calculate the crystallite size of the synthesized nHA, as shown in Table 2. It is evident that with an increased hydrothermal reaction time from 10 min to 12 h, the crystallite size marginally increases from 22.45 nm to 30.85 nm, with WP producing the smallest crystallite size. The difference in dimensions between the crystallite size and particle length arises because the crystallite size pertains to the internal atomic structure of HA, determined via XRD, whereas the particle length, observed through SEM, measures the physical dimensions of the entire HA particle, influenced by their aggregation and surface characteristics. All conditions in this experiment resulted in crystallite sizes of nHA of less than 100 nm, which is consistent with the nanoscale size of the nHA crystals found in human bone [5,39]. nHA has a large specific surface area, promoting cell proliferation and alkaline phosphatase synthesis and increasing the calcium ion concentration in the extracellular matrix [40]. Significantly, HT2 presented the highest yield of 59.27% in this study.

**Figure 3:**
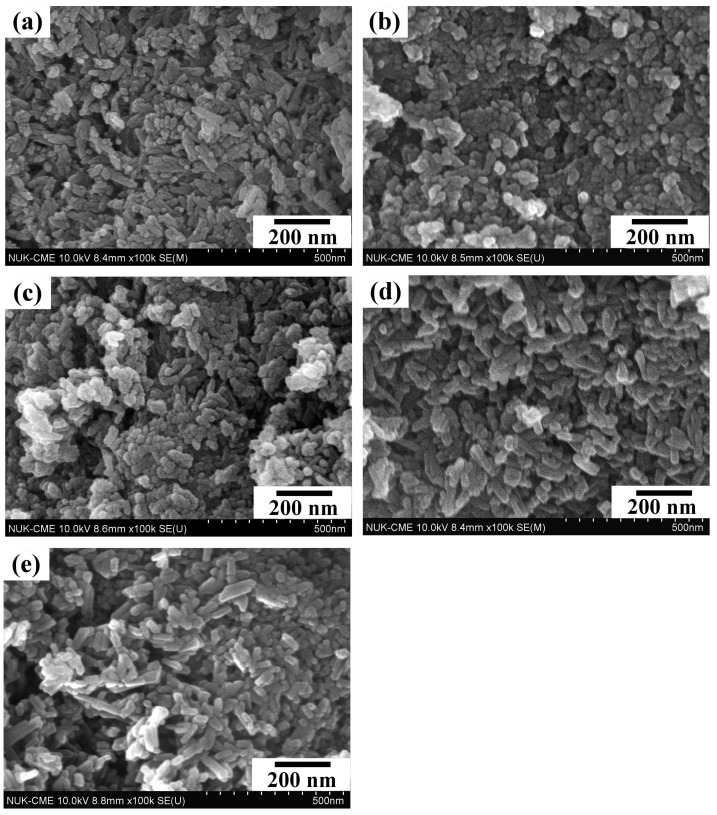
FE-SEM photographs of nHA synthesized by (**a**) wet precipitation method (WP) and hydrothermal method at various reaction times at 150 °C: (**b**) HT1—10 min; (**c**) HT2—1 h; (**d**) HT3—6 h; (**e**) HT4—12 h.

Figure 3 presents the FE-SEM photographs of the nHA synthesized via the wet precipitation method (WP) and hydrothermal method at various reaction times (HT1, HT2, HT3, and HT4) at 150 °C. The images reveal distinct morphologies for the nHA particles prepared under different conditions: WP results in rod-shaped particles, HT1 in oval-shaped particles, HT2 in a mix of oval and rod-shaped particles, and HT3 and HT4 in rod-shaped particles. Table 2 shows the length and width of the particles prepared under each condition, demonstrating that both dimensions increase with longer hydrothermal reaction times, with a more significant increase in length. Consequently, the aspect ratio (length/width) of the nHA particles increases from 1.66 to 3.98 as the hydrothermal reaction time is extended. This growth along the c-axis is typical for hexagonal close-packed (hcp) crystals of nHA, leading to rod-shaped particles [41], similar to the growth pattern of bioapatite crystals along the c-axis [42]. The XRD results corroborate this observation, with the increasing intensity of the (002) plane diffraction peak indicating growth along the c-axis and the formation of rod-shaped nHA particles.

**Figure 4:**
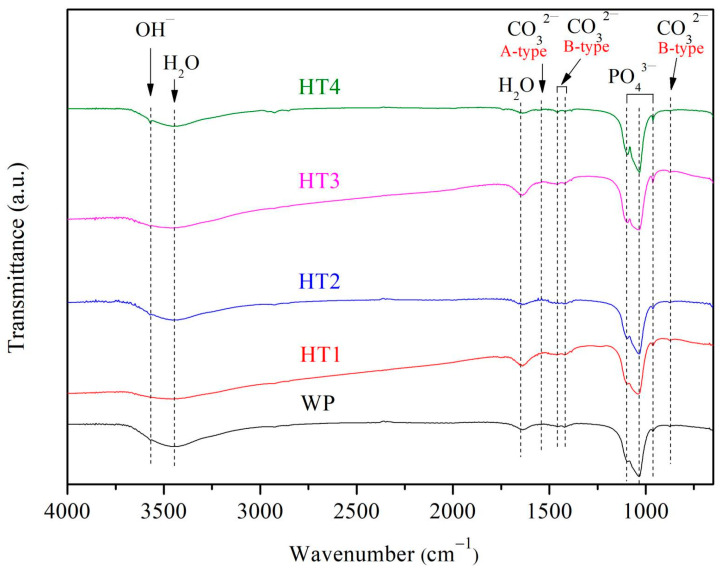
FT-IR spectra of nHA synthesized by wet precipitation method (WP) and hydrothermal method at various reaction times at 150 °C (HT1: 10 min; HT2: 1 h; HT3: 6 h; HT4: 12 h).

Figure 4 shows the FT-IR spectra of the nHA prepared under various experimental conditions. The peak at 3567 cm^−1^ corresponds to the OH^−^ group, while the bands at 1098, 1032, and 960 cm^−1^ are characteristic of PO_4_^3−^ groups in nHA. The presence of CO_3_^2−^ is observed at 870, 1418, 1460, and 1544 cm^−1^, indicating the partial substitution of OH^−^ and PO_4_^3−^ in nHA by CO_3_^2−^. The peaks at 870, 1418, and 1460 cm^−1^ correspond to B-type carbonated apatites (substitution of PO_4_^3−^ by CO_3_^2−^), while the 1544 cm^−1^ peak corresponds to A-type carbonated apatites (substitution of OH^−^ by CO_3_^2−^). The presence of CO_3_^2−^ in nHA is significant because carbonate is a component of bioapatite [43], and carbonated nHA exhibits high solubility, beneficial for resorption in vivo [44,45].

The nHA synthesized in this experiment (using HT2 as an example) primarily consists of Ca and P, with trace elements of Na (2487 ppm), Mg (1748 ppm), K (99 ppm), and Sr (535 ppm). These trace elements originate from oyster shells and offer various benefits for bone tissue growth. Mg promotes osteogenesis [46], Na aids in cell attachment and proliferation [47], K enhances collagen synthesis [48] and increases the bone density in appropriate amounts [49], and Sr-containing HA exhibits superior bioactivity [50] and is effective in treating osteoporosis [51]. Importantly, the ICP-MS analysis confirms that the oyster shell used in this experiment contains no heavy metals (As, Pb, Cd, and Hg), ensuring that the synthesized nHA is free from heavy metal contamination and meets the safety requirements (ASTM F1185-03 [52]).

Since HT2 demonstrated the highest yield among all samples prepared via the hydrothermal method, and its synthesis time effectively balanced efficiency and productivity, HT2 was chosen as the representative sample for subsequent experiments. This sample was then compared with the WP samples.

### 3.2. Bioactivity Evaluation

**Figure 5:**
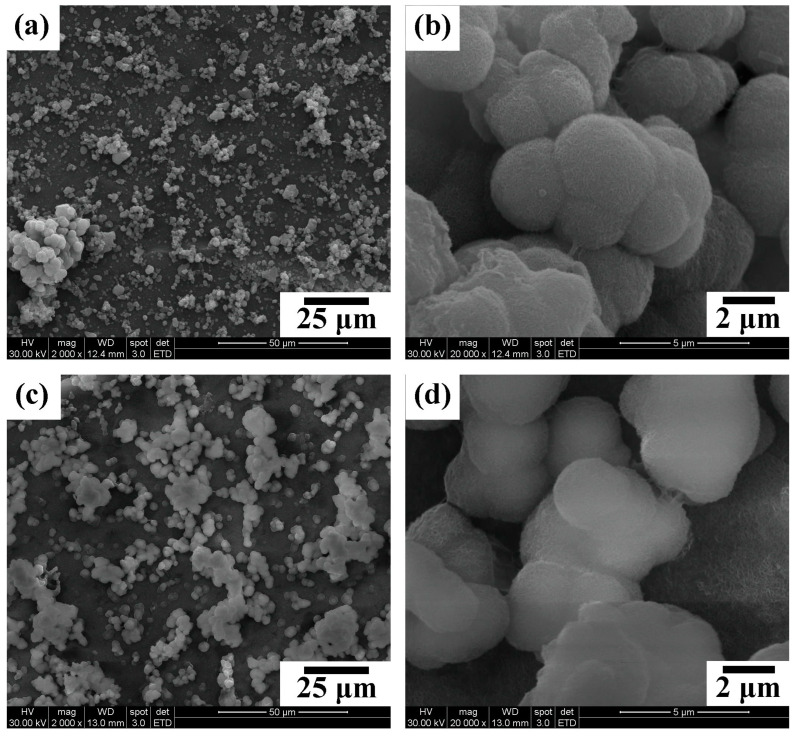
SEM photographs of the surfaces of the WP (**a**,**b**) and HT2 (**c**,**d**) samples immersed in SBF for 28 days.

Figure 5 presents the SEM photographs of the WP and HT2 samples immersed in SBF for 28 days. The images reveal that numerous spherical particles aggregate on the surfaces of both WP and HT2 samples. During the initial stage of immersion in SBF, many Ca-P particles are deposited on the sample surfaces. As the immersion time increases, these particles grow and cluster together. It was observed that the surface of HT2 was covered with more precipitates compared to WP. The high-magnification SEM images reveal that these spherical precipitates consist of numerous tiny feather-like crystals [53].

**Figure 6:**
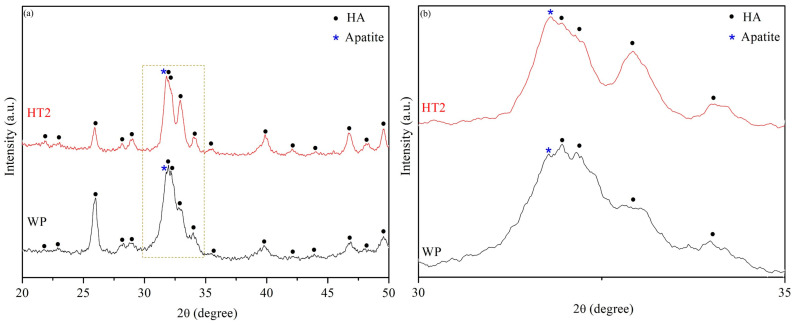
HR-XRD patterns of WP and HT2 samples immersed in SBF for 28 days. (**a**) is a wide-range XRD patterns. (**b**) is a magnified view of the dashed box area in (**a**).

The HR-XRD analysis (Figure 6) indicates that after 28 days of immersion in SBF, an apatite phase diffraction peak is clearly observed, in addition to the HA phase, for both WP and HT2. The intensity of the apatite diffraction peak is greater in HT2, indicating that the surface of HT2 has a larger quantity of apatite. This suggests that HT2 has a superior ability to induce apatite formation and demonstrates better bioactivity.

### 3.3. Cell Culture

**Figure 7:**
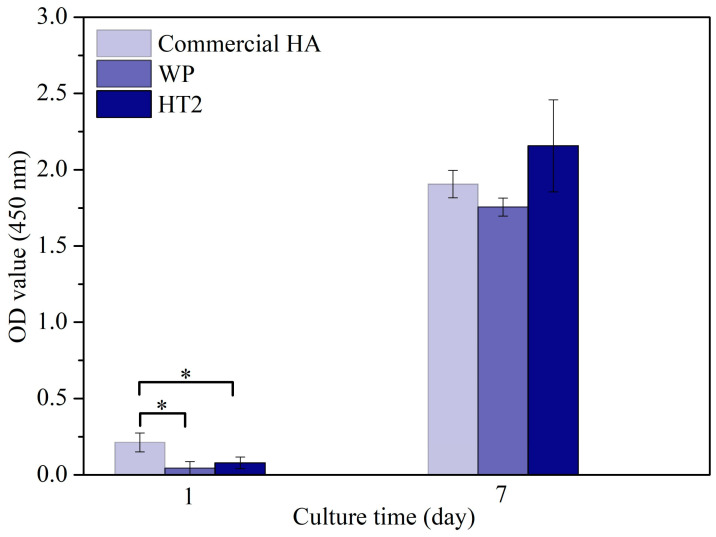
WST-1 assay of osteoblasts seeded on commercial HA, WP, and HT2 for 1 and 7 days. (An asterisk indicates statistically significant differences between groups; *p* < 0.05.).

Figure 7 presents the WST-1 assay results for HT2 and WP, compared with commercial HA, after cell culture for 1 and 7 days. The results indicate that the optical density (OD) values for all conditions were low after 1 day of culture. However, with an increase in the culture time to 7 days, the OD values rose, demonstrating that all conditions promoted cell proliferation.

**Figure 8:**
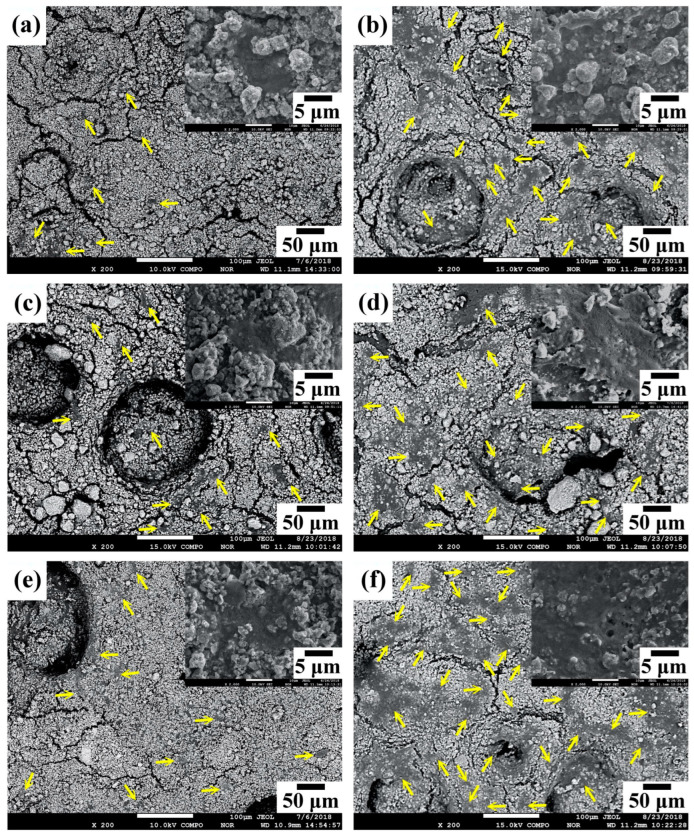
FE-SEM photographs of osteoblasts cultured with commercial HA, WP, and HT2 for 1 and 7 days, respectively. The insets show corresponding high-magnification images (upper right corner): (**a**) commercial HA, 1 day; (**b**) commercial HA, 7 days; (**c**) WP, 1 day; (**d**) WP, 7 days; (**e**) HT2, 1 day; (**f**) HT2, 7 days.

Figure 8 shows typical SEM images of the osteoblast morphology on the surfaces of commercial HA, WP, and HT2 specimens after 1 and 7 days of culture. On day 1 (Figure 8a,c,e), cells are observed adhering to the surfaces of all samples, as indicated by the arrows. The high-magnification SEM photographs reveal that the cells exhibit a flattened and spread shape, indicating good attachment. Additionally, visible filiform pseudopodia are noted. By day 7 (Figure 8b,d,f), an increased number of cells is observed on the surfaces of all samples, indicating significant cell proliferation. Among the samples, HT2 shows the largest cell coverage area.

### 3.4. Sintering Properties

**Figure 9:**
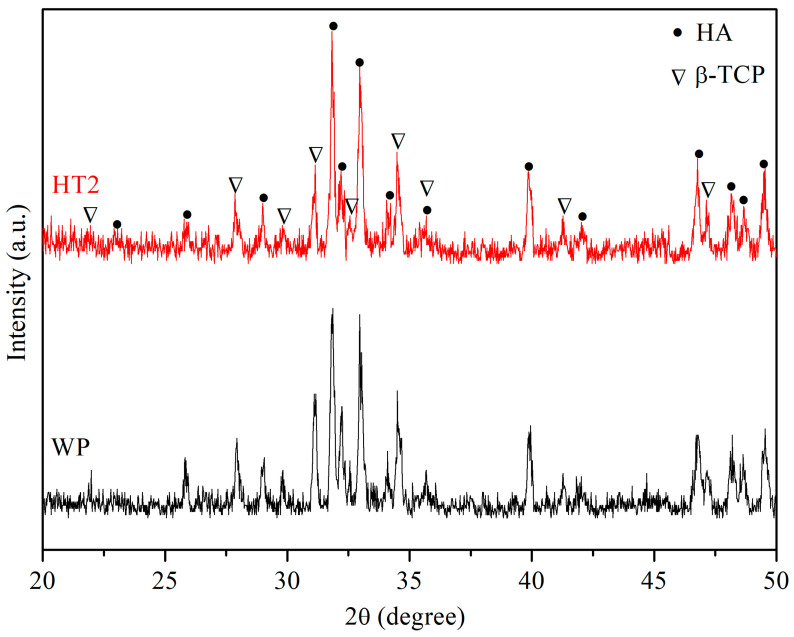
XRD patterns of WP and HT2 compacts after sintering at 1200 °C.

After WP and HT2 were press-formed and sintered at 1200 °C, the XRD patterns, shown in Figure 9, reveal that both the sintered WP and HT2 compacts are biphasic HA/β-TCP, indicating that some of the HA phase has been transformed to β-TCP. The phase composition (HA/β-TCP) of the sintered WP and HT2 compacts is shown in Table 3. The β-TCP phase content is lower in the HT2 compact, suggesting better thermal stability and reduced phase decomposition. Table 3 also shows that the microhardness of the WP and HT2 compacts is 5.26 GPa and 5.65 GPa, respectively. These values are higher than the microhardness of human tooth enamel (2.61–3.13 GPa) [54]. The microhardness is mainly influenced by the relative density, grain size, and phase composition. In this study, the relative density and grain size of the WP and HT2 compacts are quite similar, but WP has slightly higher second-phase (β-TCP) content, resulting in slightly lower microhardness than HT2.

**Figure 10:**
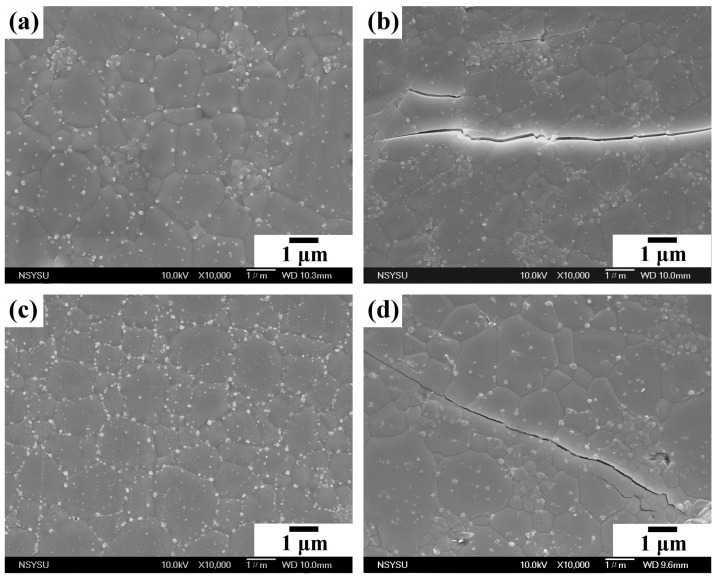
SEM photographs of sintered WP and HT2 compacts at 1200 °C and cracks generated on the surfaces of the samples after microhardness tests: (**a**,**b**) WP; (**c**,**d**) HT2.

Figure 10a,c show that the WP and HT2 samples sintered at 1200 °C are dense, with no obvious pores. The relative density of both the WP and HT2 compacts reaches approximately 94% (Table 3). The fracture toughness of the WP and HT2 compacts is 0.97 and 1.23 MPa·m^0.5^, respectively. Figure 10b,d are SEM photographs of the cracks on the surfaces of the WP and HT2 sintered samples after the microhardness test, indicating that both samples exhibit fully transgranular fracture modes. Although the fracture toughness is affected by the grain size, the grain sizes of the WP and HT2 compacts are not significantly different, resulting in similar fracture toughness values for these two sintered ceramics. Additionally, materials that exhibit intergranular fracture generally have better fracture toughness. Despite the sintered samples in this experiment fracturing via transgranular cleavage cracking, their fracture toughness values are slightly higher than that of human tooth enamel (0.94 MPa·m^0.5^) [54,55,56].

The local mechanical response of a material is crucial for various applications, especially in the biomedical field. It provides essential insights into properties such as the strength, elasticity, stiffness, and wear resistance. These characteristics directly impact the material’s performance in vivo—for instance, in orthopedic implants, where favorable local mechanical responses help to mitigate stress mismatches with tissue, thereby enhancing the long-term success and reliability of the implants [57]. Nanoscale features have a significant impact on the material properties, and they can be used to improve HA-based materials. The addition of nano-carbon materials, such as carbon nanotubes and graphene nanoflakes, enhances both the mechanical properties and biocompatibility of HA-based materials. These nanomaterials contribute to improved strength through mechanisms like second phase reinforcement and grain refinement, while also promoting cell adhesion, proliferation, and biomineralization, thereby facilitating bone regeneration and biomaterial integration in orthopedic applications [58].

## 4. Conclusions

In this study, oyster shell was utilized as a calcium source for the hydrothermal synthesis of nHA at various reaction times. The results demonstrated that the prepared nHA possessed nanoscale crystals, incorporated carbonates and trace elements, and exhibited excellent bioactivity, cell proliferation, and adhesion. These characteristics make it a promising candidate for orthopedic applications. The study identified HT2 as the most promising candidate due to its optimal morphology, high yield, and efficient processing time. HT2 demonstrated superior bioactivity and mechanical properties compared to the wet precipitation method. The use of oyster shells as a Ca source aligns with sustainable practices and provides a cost-effective alternative, highlighting the study’s contribution to developing high-quality nHA for biomedical applications. The key conclusions from this experiment are as follows.

1. Synthesis and crystallinity: Nano-grade HA was successfully synthesized using both wet precipitation and hydrothermal methods. The crystallinity of the nHA synthesized by the hydrothermal method was greater than that of the precipitation method. As the hydrothermal reaction time increased, the crystallinity of nHA also increased. Additionally, the hydrothermal time influenced the particle morphology; the nHA particles exhibited a rod-like shape when the reaction time exceeded 6 h, with the particle aspect ratio increasing over time.
2. Yield and purity: HT2 presented the highest yield of 59.27% in this study. The XRD results showed that only a single HA phase was present, with no secondary phases or residual raw materials detected.
3. Composition and trace elements: The synthesized nHA exhibited AB-type carbonation, with some OH^−^ and PO_4_^3−^ groups substituted by CO_3_^2−^. It also contained beneficial trace elements such as Na, Mg, K, and Sr, derived from the oyster shells, which promote human bone tissue growth.
4. Bioactivity and cell proliferation: After 28 days of immersion in SBF, the formation of bone-like apatite on the surfaces of the WP and HT2 samples was observed, indicating superior bioactivity. Both the WP and HT2 samples supported good cell proliferation and attachment. On day 1, the osteoblasts on the surface of HT2 exhibited a flattened and spread morphology. By day 7, the HT2 sample displayed the largest cell coverage area.
5. Mechanical properties: The WP and HT2 samples were compacted and sintered at 1200 °C for 4 h. The microhardness values were 5.26 GPa for WP and 5.65 GPa for HT2, while the fracture toughness values were 0.97 MPa·m^0.5^ for WP and 1.23 MPa·m^0.5^ for HT2. These values are higher than the microhardness (2.61–3.13 GPa) and fracture toughness (0.94 MPa·m^0.5^) of human tooth enamel, with HT2 showing superior performance.

Future research should aim to further explore the integration of HT2 into composite materials or scaffolds for biomedical applications, such as bone regeneration and drug delivery, as it holds promising prospects. Additionally, investigating the long-term stability, biocompatibility, and specific interaction mechanisms with biological systems will be crucial in translating HT2 into clinically viable solutions.

## Figures and Tables

**Table 1:** Sample code and experimental conditions under hydrothermal or vapor thermal treatment.

Sample Code	Hydrothermal	Wet Precipitation
HT1	150 °C, 10 min	–
HT2	150 °C, 1 h	–
HT3	150 °C, 6 h	–
HT4	150 °C, 12 h	–
WP	–	25 °C, 1 h

**Table 2:** Yield, crystallinity, crystallite size, and particle size (length and width) of nHA synthesized by wet precipitation method (WP) and hydrothermal method at various reaction times at 150 °C (HT1: 10 min; HT2: 1 h; HT3: 6 h; HT4: 12 h).

Conditions	Yield (%)	Crystallinity (%)	Crystallite Size (nm)	Particle Length (nm)	Particle Width (nm)	Aspect Ratio
WP	64.94 ± 2.40	30.06 ± 1.36	19.32 ± 2.36	38.28 ± 6.22	18.85 ± 3.59	2.12 ± 0.59
HT1	53.48 ± 20.98	26.04 ± 0.34	22.45 ± 2.09	40.31 ± 6.90	24.80 ± 3.61	1.66 ± 0.37
HT2	59.27 ± 2.95	36.36 ± 1.88	24.56 ± 2.09	50.61 ± 11.76	26.54 ± 6.15	1.95 ± 0.47
HT3	46.63 ± 9.00	53.04 ± 2.32	29.49 ± 2.27	82.27 ± 30.16	28.41 ± 6.18	2.99 ± 1.21
HT4	47.27 ± 13.13	54.12 ± 0.91	30.85 ± 1.50	108.75 ± 23.66	28.57 ± 6.28	3.98 ± 1.22

**Table 3:** Microhardness, fracture toughness, relative density, grain size, and crystalline phase composition of WP and HT2 compacts after sintering at 1200 °C.

	Microhardness (GPa)	Fracture Toughness (MPa·m^0.5^)	Relative Density (%)	Grain Size (μm)	Crystalline Phase Composition (HA/β-TCP)
WP	5.26 ± 0.20	0.97 ± 0.08	93.98 ± 1.37	0.81 ± 0.03	64/36
HT2	5.65 ± 0.66	1.23 ± 0.05	93.63 ± 0.69	0.85 ± 0.02	80/20

## Data Availability

The original contributions presented in the study are included in the article, further inquiries can be directed to the corresponding author.
